# Between-group behaviour in health care: gaps, edges, boundaries, disconnections, weak ties, spaces and holes. A systematic review

**DOI:** 10.1186/1472-6963-10-330

**Published:** 2010-12-07

**Authors:** Jeffrey Braithwaite

**Affiliations:** 1 Centre for Clinical Governance Research, Australian Institute of Health Innovation, University of New South Wales, Gate 11 Botany Street, Randwick, 2031, Australia

## Abstract

**Background:**

Gaps are typically regarded as a problem to be solved. People are stimulated to close or plug them. Researchers are moved to fill deficits in the literature in order to realise a more complete knowledge base, health authorities want to bridge policy-practice disconnections, managers to secure resources to remedy shortfalls between poor and idealised care, and clinicians to provide services to patients across the divides of organisational silos.

Despite practical and policy work in many health systems to bridge gaps, it is valuable to study research examining them for the insights provided. Structural holes, spaces between social clusters and weak or absent ties represent fissures in networks, located in less densely populated parts of otherwise closely connected social structures. Such gaps are useful as they illustrate how communication potentially breaks down or interactivity fails. This paper discusses empirical and theoretical work on this phenomenon with the aim of analysing a specific exemplar, the structures of silos within health care organisations.

**Methods:**

The research literature on social spaces, holes, gaps, boundaries and edges was searched systematically, and separated into health [n = 13] and non-health [n = 55] samples. The health literature was reviewed and synthesised in order to understand the circumstances between stakeholders and stakeholder groups that both provide threats to networked interactions and opportunities to strengthen the fabric of organisational and institutional inter-relationships.

**Results:**

The research examples illuminate various network structure characteristics and group interactions. They explicate a range of opportunities for improved social and professional relations that understanding structural holes, social spaces and absent ties affords. A principal finding is that these kinds of gaps illustrate the conditions under which connections are strained or have been severed, where the limits of integration between groups occurs, the circumstances in which social spaces are or need to be negotiated and the way divides are bridged. The study's limitations are that it is bounded by the focus of attention and the search terms used and there is yet to be developed a probabilistic, predictive model for gaps and how to connect them.

**Conclusions:**

Gaps offer insights into social structures, and how real world behaviours of participants in workplaces, organisations and institutions are fragile. The paper highlights the circumstances in which network disjunctures and group divides manifest. Knowledge of these phenomenon provides opportunities for working out ways to improve health sector organisational communications, knowledge transmission and relationships.

## Background

### Introduction

People tend to think that gaps are bad, representing problems to be addressed. The space causing an irritating draught under the door is a trivial example, but illustrates the point. The shortfall between your life goals and what you in fact achieve is more substantial. The chasm in many societies between the rich and the poor, and wealth disparities across first-world and underdeveloped countries are much more consequential.

In business organisations a gap analysis can be conducted when a company wishes to compare actual performance with its potential performance and then design strategies to improve. The entire enterprise of research in science and social science is predicated on the idea of systematically creating new knowledge. Investigators are moved to fill deficits in their specialty literature, i.e. to adduce new evidence in order to realise a more complete knowledge base. Social workers and police operate in parts of society where breakdowns - some social, some criminal - need to be restored.

In health care, epidemiologists, public health specialists and health economists examine unwarranted health inequities and develop methods to describe and narrow them. Health authorities want to bridge policy-practice disconnections, managers to secure resources to remedy discrepancies between poor and idealised care, and clinicians to provide services to patients across the divides of organisational silos or professional tribes.

So, people are stimulated to close or plug gaps. Yet they can be instructive. They signal to us where attention should be placed [fix the draught, achieve more life goals, tackle income differences, realise improved performance, broker societal disconnections, design a research study, address structural or clinical health sector problems]. And they thus provide opportunities to learn from them[1].

This paper examines this issue, focusing on the health system. It looks at a specific kind of ubiquitous gap in clusters of people who offer health services: the network holes, spaces and missing ties that create between-group problems and opportunities for their resolution. These groups and networks are comprised of health policymakers, managers and clinicians who cluster in formal and informal ways to provide, directly or indirectly, care to patients. They work together in relatively tight or loose, open or closed and formal or informal configurations such as communities of practice, teams, microsystems, wards, units and departments. Such groups can be analysed in terms of their network properties, that is to say their explicit, social and professional structural characteristics. Holes, spaces or missing ties can tell us a lot about the problems between groups and networks, where they have weaknesses, and where they are in need of attention. They can identify the spaces where bridge-builders - known by a range of specialist titles including mavens, liaisons, reticulists and cosmopolites - can and should operate, and they can illustrate where and how we can focus attention to address problems of communication, interaction, or teamwork.

### Aims

This brief background survey of gaps between groups and networks leads to the paper's aim: to discuss a specific exemplar, the divides in health care organisations. Despite practical and policy work in many health systems to fill spaces or bridge gaps, there are many in every complex health system, and it is valuable to study the evidence exposing them, for the insights provided. Structural holes, [2,3] disconnections[4-6] and weak or absent ties[7,8] represent fissures in groups and networks, located in less densely populated parts of more closely connected social structures. Such gaps are useful, as they illustrate how interaction is afforded across sparsely populated social terrains. They can shine a light on how communication breaks down, interactivity fails or where teamwork is weak or foundering. Structural holes are often at the boundaries of organisational silos and thus can both enable and impede inter-professional relations or inter-unit knowledge transmission[9]. The present study takes the form of a systematic analysis of the research literature. Ultimately, the longer term aim is to develop a probabilistic model based on key research findings in order to predict behaviours and find new ways of learning from spaces, addressing gaps and enhancing interactivity. In the meantime, the paper discusses and synthesises empirical and theoretical group, network or organisational culture studies on structural holes, disconnections, and weak or absent ties between stakeholders. This field illuminates threats to networked relationships and opportunities to understand the fabric of micro-organisational and micro-institutional inter-relationships.

## Methods

### Literature search

A comprehensive literature review of the topic was conducted in 2009 by interrogating the ABI/INFORM Global, CINAHL, IBSS, Medline and Psychinfo electronic literature databases since their inception, closely following a published guide to systematic reviews in health care[10]. By utilising brainstorming techniques, a mind-mapping exercise, previous research[9] and a preliminary review of the literature, the following search terms were generated: 'social boundar*', "group boundar*', 'network boundar*', 'social network boundar*', 'social group boundar*', 'liminal boundar*', 'social edge*', 'group edge*', 'network edge*', 'social network edge*', 'social group edge*', 'liminal edge*', 'social space*', 'group space*', 'network space*', 'social network space*', 'social hole*' and 'structural hole*'. Selection criteria restricted the target references, depending on database, to 'human', 'English language' and 'scholarly journals'[9].

### Literature review

Citations, abstracts and complete references where available were downloaded into Endnote X3, a bibliographic software management package. Of the 6,607 references found in the search, 6,003 remained after duplicates were removed, and these were narrowed further to 598 research articles, i.e. by excluding opinion pieces, essays, editorial contributions and other non-empirical work. The sample of references which despite the explicit criteria and systematic searching still covered a wide range of topic areas was further refined by subjecting them to scrutiny by three independent reviewers. They reviewed all papers against the inclusion criteria and met to reconcile any disagreements, discussing them until consensus was reached. Reviewers focused on assessing references specifically related to social groupings and clusters [e.g., teams, groups and networks], in social spaces [e.g., holes, edges, boundaries and gaps] and in specific places [e.g., organisations, communities, schools, churches and hospitals].

### Literature analysis

Stage one of the analysis involved a procedure whereby the references remaining which met the inclusion criteria [n = 158], were subjected to content analysis using data mining techniques employing Leximancer 3.0, an automated qualitative data analysis tool. Leximancer was employed to interrogate the downloaded abstracts to reveal the key themes and concepts, and to provide a concept map of the literature. References were then further restricted in the next stage to 2005-present apart from some papers that were considered key to the topic area, two papers were added via snowballing [n = 68], and the sample separated into health [n = 13] and general [n = 55] subsets. The final stage of the analysis was a detailed systematic review of the health subset, which is the central focus of this paper.

## Results

### Content analysis

**Figure 1:**
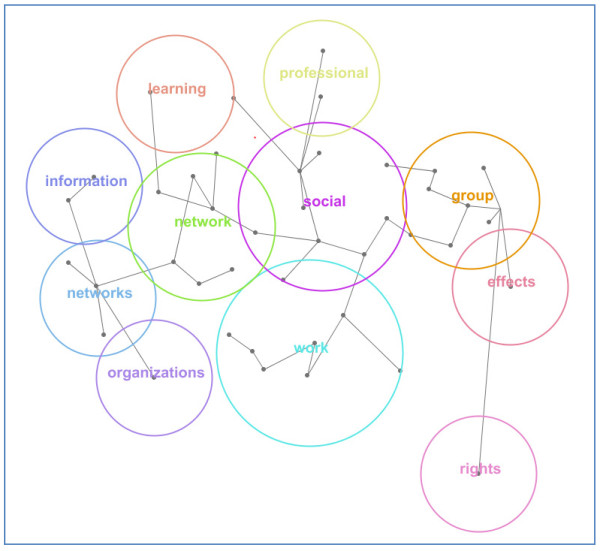
**Map of key themes in the literature on organisational social spaces, networks, boundaries and holes**.

The content analysis of the 158 research papers yielded key concepts [Table 1] with a count of the number of times the concept was used within each paper, indicating how widespread the concept is in the literature. It also provided an estimate of the relevance of the concept to the sample, indicating how linked that concept is to other concepts in the literature i.e. how related it is. The Leximancer review facilitated the production of a thematic map of the literature [Figure 1]. In the map, each circle is a theme and each dot a concept.

**Table 1:** Ranked list of key concepts and connectivity in the literature on organisational social spaces, networks, boundaries and holes

Concept	Count	Relevance	Concept	Count	Relevance
group	124	100%	management	22	18%

social	97	78%	individual	21	17%

groups	78	63%	performance	21	17%

members	52	42%	organizations	21	17%

network	47	38%	theory	20	16%

organizational	45	36%	communication	20	16%

boundaries	45	36%	different	20	16%

knowledge	44	35%	ties	19	15%

work	41	33%	processes	19	15%

networks	40	32%	structural	19	15%

results	38	31%	culture	18	15%

rights	38	31%	professional	17	14%

effects	35	28%	managers	17	14%

identity	31	25%	development	16	13%

organization	30	24%	role	15	12%

learning	29	23%	based	12	10%

employees	27	22%	using	12	10%

influence	24	19%	team	12	10%

structure	24	19%	space	12	10%

information	24	19%	used	12	10%

change	23	19%	case	3	02%

individuals	22	18%			

**Figure 2:**
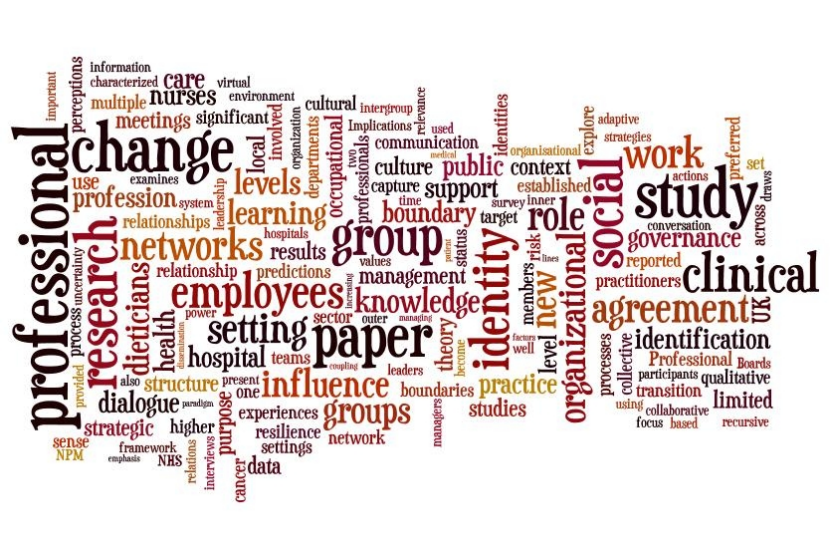
**Health wordle**.

**Figure 3:**
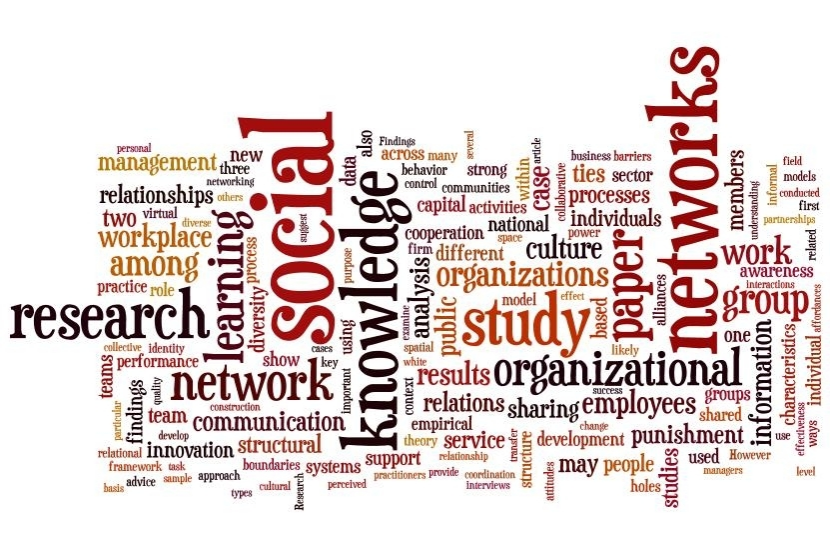
**Non-health wordle**.

The data in the table and map identify the central concepts and themes in the literature. Mostly the literature assesses *networks *in *social *context, particularly focusing on *professional*, *team *and *group work*, within *organisations*. It discusses how networks contribute to or constrain *information *and *learning*, and is interested in *network effects *and, to a lesser extent, *rights*. Associated issues include network members' *development*, *culture*, *identity *and *ties*, and there is an emphasis on network *structure*, *influence *and *performance*. When separated into health and non-health samples, analysis shows that the health literatures concentrate more on professional, clinical and care issues in contrast to the non-health literatures, focused on knowledge, organisational and social matters. Two wordle diagrams [Figure 2 Health wordle and Figure 3 Non-health wordle] providing word clouds which give prominence to frequently-occurring words in the source texts illustrate the differences http://www.wordle.net/.

### Systematic review

A systematic review of the thirteen relevant health sector studies was conducted. References were reviewed and the key features abstracted, as shown in Table 2.

**Table 2:** Characteristics of included health studies [n = 13]

Study	Methods	Participants, duration	Context	Findings	Theoretical paradigm; disciplinary perspective
Addicott (2008)	**Study design**: triangulated qualitative study. **Unit of analysis**: case studies of networks. **Method**: semi structured interviews; document analysis; ethnographic observation. **Methodological approach**: social science mixed methods.	**Number**: staff in 33 network meetings; 117 semi-structured face-to-face interviews. **Type**: provider and commissioning representatives in health care. **When**: 2002 to 2004	NHS; London, England; Cancer networks; examining mandated network structures.	Networks were under a command and control structure, under the auspices of a formalised bureaucracy. Vertical reporting rather than horizontal networking characteristics dominated. Minimal impact of these networks.	New public management theory; health services research perspective.
Braith- waite (2006)	**Study design**: ethnographic study. **Unit of analysis**: staff in acute setting clinical directorates. **Method**: observations of staff in clinical directorates in two large teaching hospitals. **Methodological approach**: examination of social structure and culture of staff and leaders.	**Number**: multiple directorate staff; four leaders in particular. **Type**: nurses and doctors. **When**: 1996 to 1999.	Australia; staff in two states.	Structurally, although the boxes on the organisational charts were altered, clinical directorates did not achieve changes to deep seated social structural arrangements and professional identity. Tribal behaviours centred on professional interests and roles continued. Relationships across the professions remained partisan and political.	Culture theory; change, particularly structural change; clinical professional organisation.
Callan et al (2007)	**Study design**: survey. **Unit of analysis**: staff in a large public teaching hospital. **Method**: administration of questionnaire survey. **Methodological considerations**: analysis of attitudinal data.	**Number**: 615 sampled respondents; 40% response rate. **Type**: health employees: doctors, nurses, allied health and administrative staff. **When**: 2005	Australia; Queensland public health system; large metropolitan teaching hospital; examining professional identity and responses to change.	Participants identified with small groups and departments rather than their organisation. Higher perceived status was associated with higher levels of job satisfaction, higher levels of openness to change, and lower levels of uncertainty. When threatened e.g. during change processes, people found their group identity is a protective mechanism.	Organisational change theory; psychological measurement of attitudes of health professionals.
Creswick et al (2009)	**Study design**: social network analysis. **Unit of analysis**: emergency department staff. **Method**: social network survey. **Methodological approach**: assessing network characteristics in a time critical area.	**Number**: 109 clinical staff in an ED. **Type**: doctors, nurses, allied health staff, administrative personnel. **When**: 2007.	Sydney, Australia; cross-sectional analysis of an ED.	There are high levels of connectedness across the ED. ED staff mostly seek help from and provide assistance to colleagues in their own profession. There are lower levels of connectedness when staff seek help for or provide advice on medication, but this is still largely within their own profession. Participants also socalised tribally, with colleagues from their own profession.	Social network theory; social structural characteristics of various staff groups in health care.
Denis et al (2001)	**Study design**: triangulated qualitative study. **Unit of analysis**: case studies of organisational change. **Method**: employee interviews; document analysis; ethnographic observation. **Methodological approach**: social science mixed methods.	**Number**: executive staff in 54 meetings; 117 interviews. **Type**: key organisational leaders and decision-makers. **When**: 1991-2001	Quebec, Canada; longitudinal change processes.	Leadership is diffused, as is power, across numerous stakeholders including external planning and funding agencies. Many forms of social structure manifest across different settings based on who dominates, how weakly or strongly coupled are teams and networks, and how change is processed. Change depends on top-group harmony; constellations of agents are fragile; change is cyclical; leadership and its relationship to those led is complex and iterative; various factors contribute to the standardization of change.	Strategic leadership process theory; organisational studies in health care.
Fortin (2008)	**Study design**: anthropological investigation. **Unit of analysis**: observations and interviews of staff and clients in two specialty paediatric services. **Method**: semi structured interviews; document analysis; ethnographic observation. **Methodological approach**: social science mixed methods.	**Number**: observations of multiple stakeholders, particularly focused on doctors; 47 medical interviews;18 case studies of patients. **Type**: doctors, other clinicians, patients and families. **When**: 2005 to 2008	Montréal, Canada; University paediatric hospital examining multi-disciplinary clinical settings (intensive care and oncology).	Emergent culture of parents, paediatric patients and clinicians is characterised by participants employing differing frames of reference. Practice differs across settings. Team meetings are spaces within which negotiated order emerges from the differing perspectives. Various roles and perspectives intermingle; there are inequalities in power and relations between clinicians, amongst clinicians groups, and between clinicians, patients and families. Doctors dominate.	Micro-cultural account: anthropological- ethnographic perspective.
Helms and Stern (2001)	**Study design**: survey. **Unit of analysis**: staff in dispersed aged care centres. **Method**: administration of questionnaire survey. **Methodological approach**: analysis of attitudinal data.	**Number**: 329 sampled respondents; 40% response rate. **Type**: staff in aged care facilities. **When**: ~1999.	United States of America; facilities distributed in a national organisation, covering 28 states.	Perceptions about cultural and sub-cultural features of organisations differed on six of 10 cultural dimensions. To some extent, attitudes vary across hierarchical levels, age, gender and ethnicity but not by staff tenure or functional area. The quest to create one big family across discrete organisational units which are part of a large chain is not likely to succeed, and homogenisation of culture via corporate encouragement and marketing strategies is problematic.	Organisational culture and sub-culture theory; health services studies focusing on aged care.
Hodges et al (2008)	**Study design**: in-depth, semi-structured interviews. **Unit of analysis**: early career nursing attitudes. **Method**: interviews until saturation achieved. **Methodological approach**: examination of socialisation in acute care.	**Number**: 11 per purposively sampled staff. **Type**: nurses with between 12 and 18 months of experience. **When**: 2002 to 2003.	United States of America; acute care settings.	New nurses undergo a socialisation process of learning the milieu (e.g. embracing the culture, acquiring a skills set); discerning their fit and identity as a nurse; and moving through their career experiences encountering pivotal points, becoming more accomplished over time. Ultimately, these themes underpin professional resilience. Key success factors include clarifying boundaries, acquiring skills, enabling accomplishments and building relationships.	Nursing socialisation theory; acute nursing profession studies.
Hotho (2008)	**Study design**: in-depth, semi-structured interviews. **Unit of analysis**: general practitioners. **Method**: purposive interviews. **Methodological approach**: investigations of GPs supportive of the formation of and leading cooperatives.	**Number**: 10 per purposively sampled staff. **Type**: GPs with leadership roles. **When**: ~2005.	NHS: Scotland; Local Health Care Co-operatives.	Even though they occupied leadership roles, participants were first and foremost clinicians rather than managers. They took on leadership roles to support professional rather than managerial interests. They at first felt idealistic about new collaborative ways of working, and self-identified as change agents, boundary-spanning medicine and management. Having a foot in each of two worlds later posed problematics for agency and professional identity.	Structuration theory; social identity theory; changing professional identities; use of scripts to narrate a meaningful story.
Matthews and Thomas (2007)	**Study design**: in-depth, semi-structured interviews. **Unit of analysis**: health services professionals. **Method**: purposive interviews. **Methodological approach**: probing how knowledge about patient safety is captured in health settings.	**Number**: nine purposively sampled staff. **Type**: seven clinicians, two managers. **When**: ~2005 to 2006.	NHS: UK; secondary care NHS trust.	People prefer oral, informal communication over other methods. Communication in complex adaptive systems is fluid, dynamical and it does not necessarily support formal bureaucratic knowledge capture, and can hinder them.	Learning theory within complex adaptive systems; phenomeno- logical health studies.
Shanley and Correa (1992)	**Study design**: triangulated qualitative study. **Unit of analysis**: case study of an organisational merger. **Method**: employee interviews; document analysis; ethnographic observation; questionnaire survey. **Methodological approach**: social science mixed qualitative- quantitative methods.	**Number**: 24 senior managers; 84 of 114 questionnaires (74% response rate) administered to decision-makers. **Type**: all top managers in two organisations. **When**: ~1990.	United States of America; an academic medical centre (the acquirer) and a community hospital (the acquired).	Decision processes in complex social-organisational environments is multi-dimensional. Dimensions of agreement include perceived agreement, actual agreement, accuracy and agreement and within one's own organisation. Personnel in organisations differ in views, and inter-group dynamics are key variables in understanding complex organisational interactivity and decision-making.	Decision theory in real world settings; acquisition theory; inter-group interaction studies.
West and Barron (2005)	**Study design**: telephone interviews of randomly sampled clinician-managers. **Unit of analysis**: views of nurse executives and physician leaders. **Method**: interviews eliciting information about participants' network boundaries. **Methodological approach**: uncovering information about alters' ties.	**Number**: 50 medical and 50 nursing interviewees, all with managerial roles. **Type**: all top managers in two organisations. **When**: ~2003.	NHS; UK; cross-NHS Sample of experienced nursing and medical managers' social relations and social boundaries.	Nurses were tied to other nurses (60% of ties) and managers; doctors were even more strongly tied to other doctors (75% of ties). Professional homophily did not extend to each other's professions. Also strongly apparent were gender and age homophily. Participants' strongest ties were geographically close, and local communication is preferentially and normally face-to-face.	Social network theory; homophily characteristics; health services studies.
Wikström (2008)	**Study design**: Semi-structured interviews of purposively sampled dieticians; analysis of organisational documentation. **Unit of analysis**: participants use of boundary work to increase their influence and power. **Method**: interviews eliciting information about participants' advocacy vis-a-vis adjacent workplace groups. **Methodological approach**: triangulated case study.	**Number**: 20 dieticians and two managers. **Type**: clinical staff. **When**: 2004 to 2005.	Sweden; University teaching hospital.	To exercise more influence, participants established a professional group, developed a narrative, advocated their competence and utility, related this to medical and nursing knowledge, developed relationships with target groups and established roles in those groups. Their influence increased as a result. Their boundary-spanning attributes included articulating competencies, emphasising collaboration, projecting social-professional identity, to some extent based on subservience to doctors and nurses. The preferred approach was not to threaten those groups. If doctors are the father and nurses the mother in a family metaphor, some dieticians describe themselves as the mistress.	Boundary roles and boundary- spanning theory; social influence theory; professional identity study.

## Discussion

In conducting a rigorous, staged systematic review to identify the key studies in the health sector about structural holes, spaces, disconnections, and weak or absent ties I note that comparatively few studies deal squarely with the gap phenomenon. The studies abstracted in Tables 1 and 2 and shown diagrammatically in Figures 1, 2 and 3 only peripherally examine social spaces, holes, boundaries, edges and poorly connected ties, instead focusing predominantly on health care groups, networks, social clusters and professional tribes. Most social and health service researchers examine groups and group behaviour rather than group boundaries or the spaces in between, despite the potential gains to be made by examining the edges and disconnections. After all, it is across these divides, holes and spaces where information is transmitted, behaviours and practices disseminate, and cultural characteristics are emulated. This means we lack understanding of this important but under-recognised dimension of our social-professional environments. Burt's work on structural holes[3,11,12] continues to offer inspiration for anyone who wishes to contribute to this area of interest, but the challenge it represents has not been taken up to a large extent, particularly in health care.

Where the results do provide evidence, they show that some groups from the bottom-up engage in boundary-spanning activities. GPs in Scotland explicitly occupied roles as leaders of Local Health Care Co-operatives but remained clinicians rather than managers in identity and outlook[13]. Dieticians in Sweden successfully ran an overt campaign to market their professional interests vis-à-vis medical and nursing colleagues and, although they occupied a modest place in the clinical pecking order, increased their visibility and influence without threatening those other groups[14]. Stakeholders in paediatric settings in Canada proceeded from their *weltanschauung*, or worldview, mobilising their particular frame of reference, and realised a negotiated order via the complex mix of their multiple perspectives, interests and needs. Inequalities in power, resources and capacity to dominate (e.g., doctors) or be subservient (e.g., patients and families) persisted[15]. Staff dispersed across many settings in the United States in a chain of aged care services did not share a common corporate culture, despite homogenisation efforts, and such differences across units were likely to persist[16]. When mandated attempts were made in the English NHS to introduce cancer networks to close gaps between provider stakeholders and encourage horizontal interactions, the hierarchical and bureaucratic influences over-rode the lateralised benefits sought by networking[17]. When push comes to shove, command and control prevails over bottom-up reform measures aimed at integration, at least in this case in the NHS.

Professional tribes and other naturally occurring divides were examined in several Australian studies. In times of change, clinicians identified more with their work group, services or department than their organisation, and this identification can act as a psychological protective mechanism[18]. In the emergency department tribalism is pronounced, and people replicate within-tribe cohesion both professionally and socially[19]. Homophily, the tendency for people with like characteristics to stick together, has a strong presence in other types of health settings. A salient example is given through an examination of NHS nursing and medical managers' professional ties. Participants' collegiate structure was highly localised. Nurses' connectedness to other nurses was at 60%, and doctors to doctors at 75%. Organisational participants do seem to prefer under most circumstances to relate to, reciprocate with or hang around others like them[20]. There is wisdom in the aphorism *birds of a feather flock together*. This seems to be reinforced through socialisation processes. During the phase of acquiring professionally-based norms, predilections and characteristics, nurses in the United States at 12-18 months of experience, for example, exhibited strong within-nursing interests. Participants were seen to spend time working out their place in the social-professional structures and cultural mileu[21].

When reviewing communication flows and decision-making in different circumstances, a British and an American study demonstrated some parallel issues. Health professionals in the UK were reported to favour oral messages and direct communication over other forms. They preferred personalised information delivered to them by other people[22]. But that is not as easy as it seems, and is time consuming to accomplish, particularly in times of stress. Decision-making and communication are complex at the best of times. During a merger of two hospitals, differences in outlook and perspective became apparent, and added to the political and communicative pressures[23].

Studies of organisational change in Scotland, [13] Australia, [24] Canada, [25] America, [16] Sweden[14] and Britain[20] bear out the proposition that identification with one's primary group or profession is very strongly held, yet cross-departmental, cross-group, cross-professional communication, collaboration and interaction are crucial in creating more pluralist, informed and supportive workplaces. The benefits from understanding others' perspectives, knowledge and skills are frequently latent but considerable. It follows from this analysis that the quest to bridge gaps, fill structural holes, connect groups, services and professions, and strengthen weak ties or remedy absent ties in network structures is perennial. Yet the ways to do this remain unclear, and lacking a sound evidentiary basis.

There are hints at how to succeed. Running a concerted campaign to improve one group's utility to another, by drawing up an extended narrative of what one is offering, optimised the Swedish dieticians' power and influence[14]. Appreciating the other group's point of view, [23] and relating to their needs, [25] is another strategy calculated to help win someone over. Communicating with others verbally, [22] through personalised messages on their own terms, is expected to be useful as is recognising that the other group's, network's or profession's identity is deep-seated, [13,18-20,24] and not likely to be negotiable or tradeable. And we should recognise Burt's point: *"Structural holes are entrepreneurial opportunities for information access, timing, referrals, and control"*[12].

There are study limitations. This project is a product of its focus on a subset of the literature on gaps related to organisations, and as is the case with all systematic reviews, the findings are restricted to the search terms employed. There is no probabilistic, predictive model of gaps, how they manifest, or what can be done about them to close disjunctions or improve communication or interactivity across boundaries and spaces.

## Conclusions

Whether we are dealing with naturally-occurring ones, or those mandated by people in positions of authority, networks and groups tend towards segregation. Barriers, gaps and disconnections between clusters of people are the norm. Within-group myopia predominates. The examination of these case examples, surveys and studies was conducted in order to understand where relations are weak, and to explicate the range of opportunities for improved social relations that understanding structural holes, spaces, disconnections and absent ties affords. A principal point is that these kinds of gaps shed light on the circumstances under which ties have been severed, the limits of connectivity between groups, [5] and social space across which people need to negotiate[15]. This phenomenon can be a signifier of lack of trust in the nearby network, [26] highlighting instances where people have stopped communicating or have withdrawn from dissatisfying or discouraging relationships[3]. Most settings will need to provide opportunities for people with roles such as bridges, mavens, liaisons, reticulists and cosmopolites to make meaningful and sustainable connections if organisations and institutions are to become more joined up at their boundaries.

Gaps offer insights into social structures, are interesting phenomenon, and afford windows into how real world behaviours of participants in workplaces, organisations and institutions are fragile at the edges. In discussing the circumstances in which network disjunctures occur, and how and when, we need to remember the goal is to formulate new ways of improving health sector organisational communications, knowledge transmission and relationships across pre-existing divides.

## Competing interests

The authors declare that they have no competing interests.

## Pre-publication history

The pre-publication history for this paper can be accessed here:

http://www.biomedcentral.com/1472-6963/10/330/prepub
